# Multidrug resistant *Klebsiella Pneumoniae* reservoir and their capsular resistance genes in cow farms of district Peshawar, Pakistan

**DOI:** 10.1371/journal.pone.0282245

**Published:** 2023-02-27

**Authors:** Saddam Saddam, Muddasir Khan, Muhsin Jamal, Sadeeq Ur Rehman, Petr Slama, Pavel Horky

**Affiliations:** 1 Department of Microbiology, Abdul Wali Khan University Mardan, Mardan, Pakistan; 2 Centre of Biotechnology and Microbiology, University of Peshawar, Peshawar, Pakistan; 3 College of Veterinary Sciences and Animal Husbandry, Abdul Wali Khan University, Mardan, Pakistan; 4 Department of Animal Morphology, Physiology and Genetics, Faculty of AgriSciences, Mendel University in Brno, Brno, Czech Republic; 5 Department of Animal Nutrition and Forage Production, Faculty of AgriSciences, Mendel University in Brno, Brno, Czech Republic; Beni Suef University Faculty of Veterinary Medicine, EGYPT

## Abstract

*Klebsiella pneumoniae* is a major zoonotic pathogen that causes a variety of severe illnesses as well as mastitis. The distribution of mastitis-causing *K*. *Pneumoniae* and its virulence factors vary by country and geographical location. The present study aimed to find out the occurrence of Multidrug-resistant (MDR) *K*. *Pneumoniae* and their capsular resistance genes which were undocumented previously in cow farms of district Peshawar, Pakistan. A total of 700 milk samples from symptomatic mastitic cows were screened for MDR *K*. *Pneumoniae*. Furthermore, the characterization of capsular resistance genes was done by molecular techniques. Among these samples, *K*. *pneumoniae* was found 180/700 (25.7%), while MDR *K*. *pneumoniae* was found 80/180 (44.4%). The antibiogram analysis revealed high resistance to Vancomycin (95%) while highly sensitive to Ceftazidime (80%). The distribution of capsular genes shows the most common serotype K2 gene 39/80 (48.7%), followed by serotype K1 gene 34/80 (42.5%), serotype K5 17/80 (21.2%), and serotype K54 13/80 (16.2), respectively. Moreover, the co-occurrence of serotypes K1+K2 was found at 11.25%, KI+K5 was 05%, K1+K54 was 3.75%, and K2+K5 was 7.5%, respectively. A statistically significant association (p ≤ 0.05) was found between predicted and discovered *K*. *pneumoniae* values. In conclusion, the presence of MDR *K*. *pneumoniae* in combination with capsular genes may be a possible threat to dairy farm animals and humans in Peshawar, Pakistan. It may give us special attention to follow up on hygienic practices in livestock management.

## Introduction

Cow mastitis is one of the most common infections in dairy cows. It exclusively affects the dairy cow’s reproductive cycle and maternity, resulting in decreased milk output and quality. It also raises treatment costs and creates significant economic losses in dairy sectors worldwide [1]. Mastitis causes $35 billion in global economic losses each year, with the US dairy industry losing $2 billion [2, 3]. Furthermore, mastitis can endanger human and animal health by transferring antibiotic-resistant bacteria and causing food poisoning [2]. *Klebsiella pneumoniae* is a major environmental pathogen that causes mastitis, as well as a zoonotic pathogen that may cause a variety of severe illnesses [4, 5]. Numerous properties involved in *K*. *pneumoniae* pathogenesis have been identified, like virulence factors, but their capability in toxicity and drug resistance remains unknown [6].

The pathogenic mechanism of *K*. *pneumoniae* is also determined by virulence factors. Capsular, iron carriers, pili, and lipopolysaccharide (LPS) have all been associated in *K*. *pneumoniae* adhesion, invasion, and proliferation [7, 8]. The capsular can stop *K*. *pneumoniae* from being recognized by the host immune system through immune escape mechanisms like inhibition of early inflammatory response, anti-phagocytosis, inhibition of dendritic cell maturation, and neutralization of antimicrobial peptides to lessen the body’s immune response [8]. These bacteria may take iron from the host via four siderophores, including salmochelin, aerobactin, yersiniabactin, and enterobactin for metabolism and infection [9]. As the researches are still in its early stages, *K*. *pneumoniae* pathogenesis has long been linked to four key components *K*. *pneumoniae* capsule antigens, adherence factors, lipopolysaccharides, and Siderophores. The virulence factor for *K*. *pneumoniae* was capsular polysaccharide (K antigen). The sparkling and mucoid presence *of K*. *pneumoniae* settlements on agar plates due to this antigen shapes a thick hydrophilic case. At least 77 K antigen serotypes have been recognized to date, referred to as K1, K2, etc. The K antigens assume a significant part in shielding cells from opsonophagocytosis and serum killing [10].

Mastitis incidence varies by country and geographical location because of variances in legislation, veterinarian and laboratory facilities, and farmer management techniques. Therefore, the present study was designed to find out the occurrence of multi-drug resistant (MDR) *K*. *Pneumoniae* in cow farms and their comparative analysis with capsular resistance genes in the district of Peshawar, Pakistan.

## Materials and methods

### Sample collection and isolation of *K*. *pneumoniae*

The animal study protocol was approved by the Institutional Review Board of Abdul Wali Khan University Mardan, Pakistan. The study was performed in various areas of Peshawar, Pakistan, located at 34.0151°N, 71.5249°E (**S1 Table)**. The samples were collected from symptomatic mastitis cows in sterile falcon tubes after discarding a few drops of milk on verbal permission from the dairy farm management. All samples were sealed with air-tight closing to prevent contamination and were processed in the microbiology Laboratory, Abdul Wali Khan University Mardan, Pakistan. The milk samples were inoculated with Luria Bertani broth medium for 24 hours at 37°C. After incubation, the medium was subcultured on *K*. *pneumonia* specific media Simmons citrate agar (SCA) followed by Rodrigues et al. [11] method. Further confirmation was done by gram staining and string test. Molecular confirmation of *K*. *pneumoniae* isolates was done by Polymerase chain reaction (PCR) using their specific 16s RNA primer (**Table 1**) [12].

**Table 1:** Primers used for molecular characterization of MDR *K*. *pneumoniae* capsular resistance genes.

Gene Name	Primers	Amplification	Fragment Size	References
16s RNA	F-ATTTGAAGAGGTTGCAAACGAT	58°C	260bp	Liu et al. [12]
	R- TTCACTCTGAAGTTTTCTTGTGTTC			
K1	F- GGTGCTCTTTACATCATTGC	58°C	1283bp	Yeh et al. [13]
	R- GCAATGGCCATTTGCGTTAG			
K2	F- CAACCATGGTGGTCGATTAG	58°C	531bp	Yu et al. [14]
	R- TGGTAGCCATATCCCTTTGG			
K5	F- TGGTAGTGATGCTCGCGA	55°C	280bp	Turan et al. [15]
	R- CCTGAACCCACCCCAATC			
K54	F- CATTAGCTCAGTGGTTGGCT	55°C	881bp	Turan et al. [15]
	R- GCTTGACAAACACCATAGCAG			

### Detection of MDR *K*. *pneumoniae*

Mueller-Hinton agar was used for antibiotic susceptibility of *K*. *pneumoniae* isolates for commonly used 14 different antibiotics discs (**Table 2**), using the standard Kirby-Bauer disk diffusion technique [16]. Multi antibiotic resistance (MAR) index was calculated for MDR *K*. *pneumoniae* determination, as described by Ayandele et al. [17]. The following formula used for the MAR index:

MAR Index = a/b (a = number of antibiotics to which isolate resistant, b = total antibiotics tested)

**Table 2:** List of antibiotic discs used for the detection of MDR *K*. *pneumoniae*.

Antibiotic Discs	Abbreviations	Concentrations
Amoxicillin-clavulanic acid	AMC	10 μg
Amoxicillin	AML	10 μg
Ceftazidime	CAZ	30 μg
Fusidic Acid	FD	10 μg
Chloramphenicol	C	30 μg
Ciprofloxacin	CIP	05 μg
Levofloxacin	LEV	30 μg
Sulfamethazine	SXT	23.75 μg
Cefepime	FEP	30 μg
Vancomycin	VA	30 μg
Amikacin	AK	30 μg
Gentamycin	CN	10 μg
Tetracycline	TE	30 μg
Imipenem	IMP	10 μg

### Detection of capsular resistance genes

Total nucleic acid from the MDR *K*. *pneumoniae* isolates was extracted through WizPrepTM gDNA Mini Kit (Seongnam-si, Gyeonggi-do, 13209, Republic of Korea). Molecular characterization of four (04) capsular encoding genes; serotype K1, serotype K2, serotype K5, and serotype K54 was performed through PCR by using gene-specific primers, as given in **Table 2**. PCR Primer-specific fragments were visualized in 01% agarose gel electrophoresis using Gel Doc^TM^EZ (BIO-RAD).

### Data analysis

The expected and detected *K*. *pneumoniae* values links were analyzed through a chi-square test by SPSS version 20 and p ≤ 0.05 values were considered statistically significant.

## Results

A total of 700 samples were collected from symptomatic mastitis cows. All samples were cultured on specific media SCA for the isolation of *K*. *pneumoniae*. After the appearance of a yellowish colony, gram-negative and string test positive, the bacterium was assumed as *K*. *pneumoniae*. They were confirmed by molecular analysis, the 16s RNA gene size 260bp was conformed as *K*. *pneumoniae* (**S1 Fig in S1 Raw images**). Among the total collected samples, *K*. *pneumoniae* growth was observed in 180 (25.7%).

### MDR *K*. *pneumoniae*

**Fig 1:**
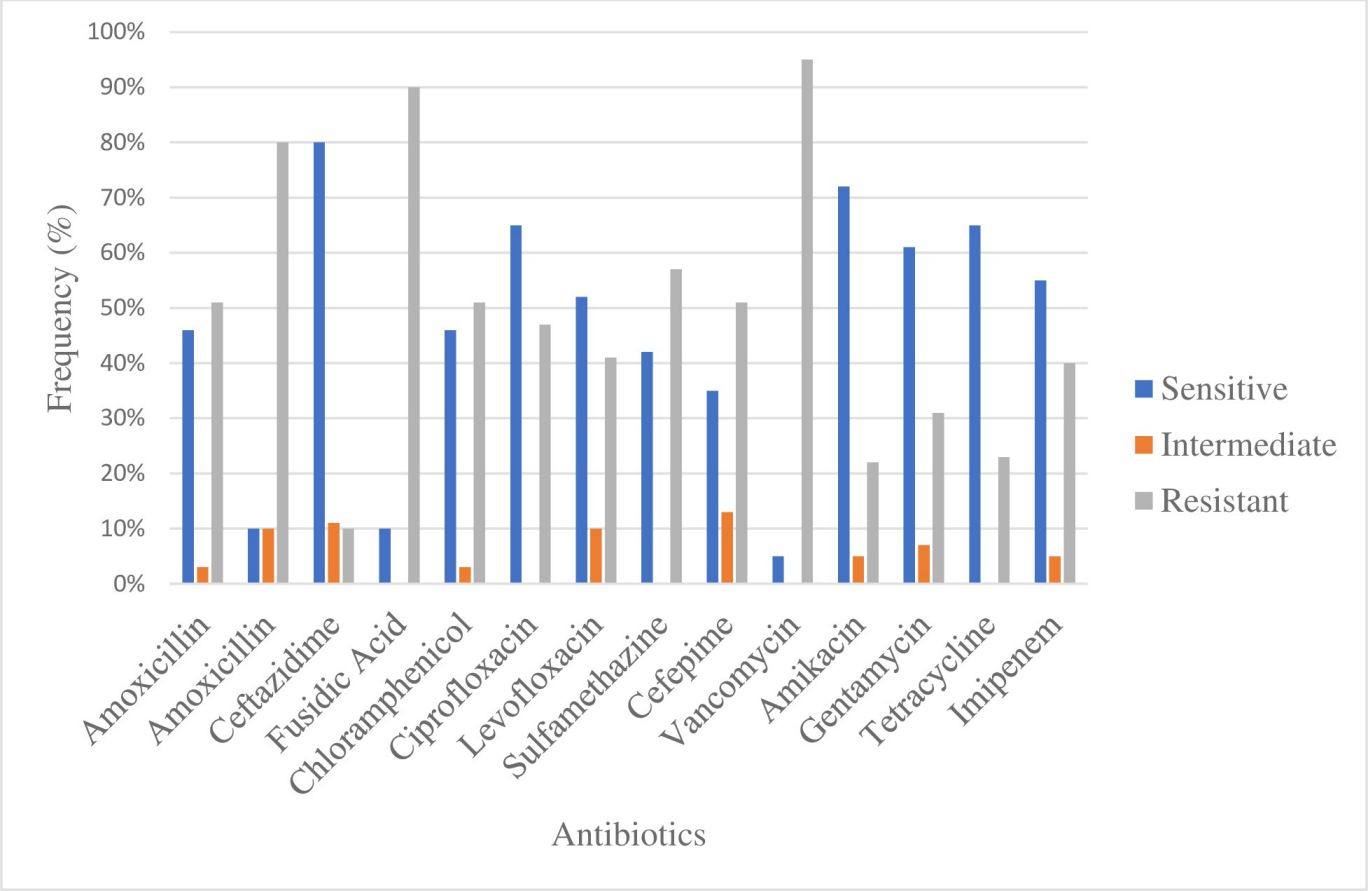
Antibiotics sensitivity pattern of MDR *K*. *pneumoniae*.

All isolated *K*. *pneumoniae* colonies were subjected for the screening of their sensitivity pattern. The MDR *K*. *pneumoniae* occurrence was observed n = 80 (44.4%) with observed MAR ranges from 0.21–0.92. The antibiogram analysis revealed high resistance to Vancomycin (95%) while highly sensitive to Ceftazidime (80%). The other antibiotic’s antibiogram results were presented in **Fig 1** and **S2 Table**.

### Occurrence of capsular genes

**Fig 2:**
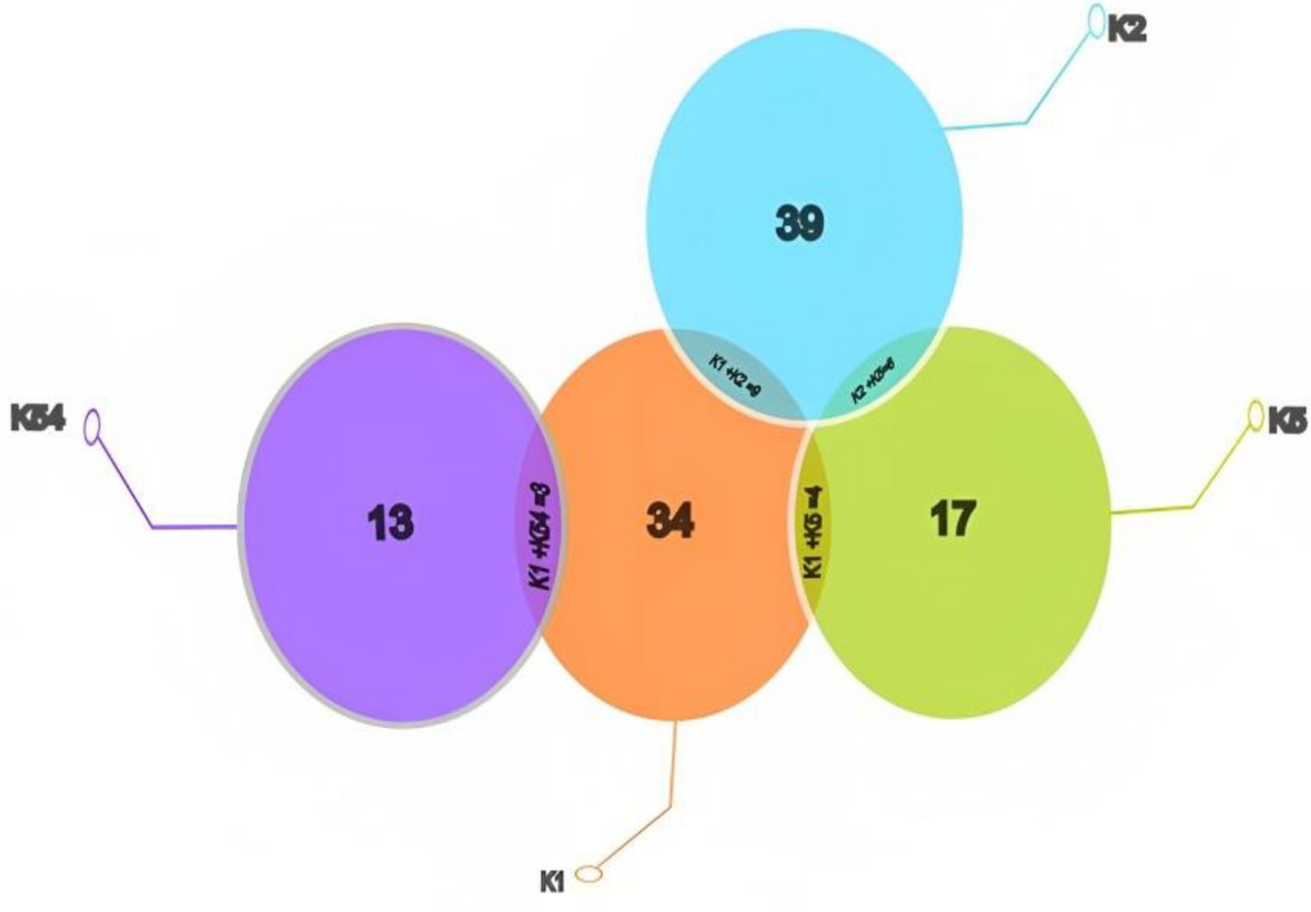
Occurrence of MDR *K*. *pneumoniae* capsular resistance genes in district Peshawar, Pakistan.

MDR *K*. *pneumoniae* was further screened for the occurrence of capsular genes. The obtained electrophoretogram of capsular resistance genes was presented in **S2-S5 Figs in S1 Raw images**. The high occurrence was found of serotype K2 gene n = 39 (48.7%) followed by serotype K1 gene n = 34 (42.5%), serotype K5 n = 17 (21.2%), and serotype K54 gene n = 13 (16.2), respectively. Moreover, the co-occurrence of serotypes K1+K2 was detected n = 09 (11.25%), KI+K5 was n = 04 (05%), K1+K54 was n = 03 (3.75%), and K2+K5 was n = 06 (7.5%), respectively (**Fig 2**). Further area wise distribution of MDR *K*. *pneumoniae* with their capsular genes was presented in **Table 3**.

**Table 3:** Overall area-wise distribution of MDR *K*. *pneumoniae*, phenotypes to the antibiotics, and capsular resistance genes in district Peshawar, Pakistan.

Areas of Peshawar	Sample Type	Total Samples	*K*. *P* Positive	MDR	Resistance Phenotypes to the Antibiotics	No. of Capsular Genes Detected			
						K1	K2	K5	K54
Arbab landi	Milk	34	8	5	FEP,VA,C, AML,FD, TE,AK,CAZ,SXT	2	2	1	1
Phushtakhara	Milk	18	9	3	AML,FD,SXT,FEP,VA,C,IMP,LEV,TE	1	2	1	0
Surband	Milk	30	7	4	VA,C,TE,AML,FD,IMP,CN,LEV FEP	1	2	1	1
Sanghu	Milk	18	4	3	AML,FD,SXT,FEP,VA,C,IMP,LEV,TE	1	2	0	1
Masho khel	Milk	24	6	4	AML,FD,SXT,FEP,VA,C,TE,AK,CAZ	2	2	1	0
Tehkal Payan	Milk	20	8	3	FD,AML,SXT,FEP,VA,C,IMP,LEV,TE	1	2	1	0
Supaid Dairy	Milk	30	6	4	AML,FD, LEV,VA,C,TE,AK,CAZ	2	2	1	0
Bazid Khel	Milk	26	4	3	FD,SXT,AML,FEP,VA,C,TE,IMP,CN,LEV	1	1	2	0
Sheikh muhammadi	Milk	22	4	4	AML,FD,SXT,FEP,VA,C,LEV,CAZ,AK	2	2	1	0
Palusai	Milk	18	7	1	FD,SXT, AML,FEP,VA,C,TE,CIP	0	1	0	1
Gharib Abad	Milk	18	5	4	FD,SXT, AML, FEP,VA,C,TE,AK,CAZ	2	2	0	0
Urmar	Milk	22	6	3	AML,FD,SXT,FEP,VA,C,	2	1	0	1
Taj Abad	Milk	24	9	6	FD,AML,LEV,CAZ,AK,SXT,FEP,VA,C	3	2	0	2
Bata Tal	Milk	16	4	3	AML,TE,IMP,CN,LEV,FD,SXT,FEP,VA,C	0	2	1	1
Shahab khel	Milk	24	9	2	FD,AML,SXT,FEP,VA,C,LEV,CAZ,AK	2	2	0	0
Mattani	Milk	24	4	2	FD, AML,SXT,FEP,VA,C,TE,AK,CAZ	0	1	1	1
Bara Gate	Milk	22	7	2	FD, AML,SXT,FEP,VA,TE,AK,CAZ,C	0	0	1	1
Custom chowk	Milk	22	7	2	AML,LEV,CAZ,AK,FD,SXT,FEP,VA,C	1	2	0	0
Nothia	Milk	22	2	2	LEV,TE,FEP,VA,C,AML,FD,SXT,IMP,	0	1	1	1
Abdhara	Milk	18	4	1	AML,FD,SXT,FEP,VA,C,TE,AK,CAZ	1	1	0	0
Chamkani	Milk	38	9	3	IMP,CN, AML,FD,SXT,FEP,VA,C,TE	1	1	1	0
Hazar Khwani	Milk	22	4	3	AML,FD,SXT,FEP,VA,C,TE,AK,CAZ	2	1	0	1
Duran Pur	Milk	22	5	2	C,LEV,CAZ,AK,AML,FD,SXT,FEP,VA	1	0	1	1
Jawad Tower	Milk	24	5	2	AML,FD,SXT,FEP,VA,C,TE,AK,CAZ	1	1	0	0
Phundu	Milk	48	15	4	IMP,LEV,TE, AML,FD,SXT,FEP,VA,C	2	1	1	0
Khazana	Milk	16	5	1	AML,FD,SXT,FEP,VA,C,TE,AK,CAZ	0	1	0	0
Farid Abad	Milk	20	4	1	SXT,FEP,TE,AK,CAZ,VA,C, AML,FD	0	0	1	0
Gul Bahar	Milk	32	9	2	AML,FD, IMP,LEV,TE,SXT,FEP,VA,C	2	1	0	0
Bakhshi Pul	Milk	26	4	1	C,TE,CAZ AML,FD,SXT,FEP,VA	1	1	0	0

The abbreviations of antibiotics were shown in **Table 2**

### Statistical results

The chi-square test demonstrated a statistically significant association between predicted and discovered *K*. *pneumoniae* values, validating our null hypothesis (p ≤ 0.05).

## Discussion

One of the dairy cow illnesses that pose the greatest economic challenges is mastitis, particularly for small-scale producers across the world. Mastitis is typically more prevalent in nations with underdeveloped dairy industries and lax udder cleanliness [18]. In the present study the symptomatic mastitic milk samples from cows in Peshawar, Pakistan were collected. The samples were cultured for the estimation of occurrence frequency and isolation of MDR *K*. *pneumoniae*. Furthermore, the capsular genes K1, K2, K5, and K54 occurrence incidence were also determined among the isolated MDR *K*. *pneumoniae*.

*Klebsiella* mastitis is a significant problem in the USA [18]. In Bangladesh, *Klebsiella* spp. was found in 62.5% of mastitic milk [19] while in India found 20% to 24% in different studies [20, 21]. In the present study, among the total 700 collected milk samples of cows, *K*. *pneumoniae* incidence was found 25.7%. In contrast to our study, a recent study in Pakistan indicated 7.9% of in Peshawar, Khyber Pakhtunkhwa among cows [22], 8% incidence due to *K*. *pneumoniae* in Punjab buffaloes [23], and 11% in Sindh province among buffaloes [24]. The current study variation may be due to specie, veterinarian facilities, and farmer management techniques.

Antibiotic overuse contributes to the development of multi-drug resistant (MDR) organisms. The pace of MDR *Klebsiella* spp., growth is accelerating daily. Globally, concerns including the rise of MDR bacteria, the use of antibiotics to treat them, and zoonotic diseases are considered critical [19]. The MAR index is determined by dividing the number of antibiotic isolates that are resistant by the total number of antibiotics the organism has been to expose [17]. In the current study, MDR *K*. *pneumoniae* was found at 44.4% with a MAR range from 0.21–0.92. These results are in agreement with previous studies of Salauddin et al. [19], who reported a 62.5% incidence of MDR *K*. *pneumoniae* and Osman et al. [18] reported 40%, while in contrast to our study, the Yang et al. [25] reported 9.78% MDR *K*. *pneumoniae*. The present study antibiogram analysis of cow milk isolated MDR *K*. *pneumoniae* showed high resistance to Vancomycin, Fusidic acid, Amoxicillin, Sulfamethazine, and Chloramphenicol, while highly sensitive to Ceftazidime, Ciprofloxacin, Levofloxacin, Amikacin, Gentamycin, Tetracycline, and Imipenem. These results are mostly similar to the previously reported study of Ali et al. [22]. The variation in the resistance profile of the present study is due to the overuse of these antibiotics for the treatment of mastitis as well as infected milk consumers.

Capsular and other virulence components are crucial to *K*. *pneumoniae*’s pathogenic process. Through immune escape mechanisms like anti-phagocytosis, inhibition of early inflammatory response, neutralization of antimicrobial peptides to lessen the body’s immune response, and inhibition of dendritic cell maturation, capsular can stop *K*. *pneumoniae* from being recognized by the host immune system [8]. Herein this study, the most common capsular gene was found K2 gene (48.7%), while serotype K1 gene was found at 42.5%, followed by serotype K5 (21.2%), and serotype K54 (16.2%). In agreement with these findings, Osman et al. [18] also reported the distribution of the K1 gene (66.7%) and K2 gene (55.6%), while Gao et al. [26] reported the K5 gene (10%) and K54 gene (8%).

## Conclusions

In conclusion, the current study results showed the high occurrence of MDR *K*. *pneumoniae* in cow farms of district Peshawar Pakistan. We must pay particular care since *K*. *pneumoniae* present in mastitic milk has capsular resistance genes, which might lead to clinical infections in cows and those who consume milk. The development of veterinary diagnostic labs for routine animal screening and antibiogram analysis is currently necessary to combat empirical antibiotic usage and resistance to certain antibiotic groups. It must be ensured follow up the hygiene practices in dairy farms.

## Supporting information

S1 Table Study area of district Peshawar, Pakistan with its geographical coordinates from where cow farms located. (DOCX)

S2 Table Antibiotics sensitivity pattern of MDR *K*. *pneumoniae*. (DOCX)

S1 Raw images (PDF)
