# Supplementary material for: Multidrug resistant Klebsiella Pneumoniae reservoir and their capsular resistance genes in cow farms of district Peshawar, Pakistan
Source: PLoS One. 2023 Feb 27;18(2):e0282245. doi: 10.1371/journal.pone.0282245 (PMC9970052; doi:10.1371/journal.pone.0282245)
Supplement: S1 Table — (DOCX) [file pone.0282245.s001.docx]

**Table S1.** Study area of district Peshawar, Pakistan with its geographical coordinates from where cow farms located.

| **Area** | **Geographical coordinates** |
| --- | --- |
| Arbab landi | 33.977910°N, 71.529213°E |
| Phushtakhara | 33.9536°N, 71.4923°E |
| Surband | 33.9365° N, 71.4863° E |
| Sanghu | 33.9347° N, 71.4745° E |
| Masho khel | 71.5041° N, 33.9069° E |
| Tehkal Payan | 34.0183° N, 71.5279° E |
| Supaid Dairy | 33.982° N, 71.487° N |
| Bazid Khel | 33.9574° N, 71.5516° E |
| Sheikh muhammadi | 33.932° N, 71.519° E |
| Palosai | 34.0278° N, 71.4884° E |
| Gharib Abad | 34.0224° N, 71.5958° E |
| Urmar | 33.9578° N, 71.7296° E |
| Taj Abad | 33.9845° N, 71.4618° E |
| Bata Tal | 33.9221° N, 71.4626° E |
| Shahab khel | 33.9262° N, 71.4985° E |
| Mattani | 33.7989° N, 71.5546° E |
| Bara Gate | 33.9804° N, 71.5229° E |
| Custom chowk | 33.9732° N, 71.5159° E |
| Nothia | 33.9936° N, 71.5464° E |
| Abdhara | 33.9787° N, 71.5042° E |
| Chamkani | 34.0059° N, 71.6481° E |
| Hazar khwani | 33.9927° N, 71.6066° E |
| Duran Pur | 34.0285° N, 71.6286° E |
| Jawad Tower | 34.0030° N, 71.5002° E |
| Phundu | 33.9873° N, 71.6406° E |
| Khazana | 34.0729° N, 71.5935° E |
| Farid Abad | 34.5281° N, 73.4758° E |
| Gul Bahar | 34.012495° N, 71.60355° E |
| Bakhshi pul | 34.0616° N, 71.5883° E |
