# Supplementary material for: Multidrug resistant Klebsiella Pneumoniae reservoir and their capsular resistance genes in cow farms of district Peshawar, Pakistan
Source: PLoS One. 2023 Feb 27;18(2):e0282245. doi: 10.1371/journal.pone.0282245 (PMC9970052; doi:10.1371/journal.pone.0282245)
Supplement: S2 Table — (DOCX) [file pone.0282245.s002.docx]

**Table S2.** Antibiotics sensitivity pattern of MDR *K. pneumoniae*

| **Antibiotic Disc** | **Abbreviation** | **Sensitive** | **Percentage**  **(%)** | **Resistant** | **Percentage**  **(%)** | **Intermediate** | **Percentage**  **(%)** |
| --- | --- | --- | --- | --- | --- | --- | --- |
| Amoxicillin + Clavulanate Acid | AMC | 37/80 | (46%) | 41/80 | (51%) | 2/80 | (3%) |
| Amoxicillin | AML | 8/80 | (10%) | 64/80 | (80%) | 8/80 | (10%) |
| Ceftazidime | CAZ | 64/80 | (80%) | 8/80 | (10%) | 9/80 | (11%) |
| Fusidic Acid | FD | 8/80 | (10%) | 72/80 | (90%) | 0/80 | (0%) |
| Chloramphenicol | C | 37/80 | (46%) | 41/80 | (51%) | 2/80 | (3%) |
| Ciprofloxacin | CIP | 52/80 | (65%) | 38/80 | (47%) | 0/80 | (0%) |
| Levofloxacin | LEV | 42/80 | (52%) | 30/80 | (41%) | 8/80 | (10%) |
| Sulfamethazine | SXT | 34/80 | (42%) | 46/80 | (57%) | 0/80 | (0%) |
| Cefepime | FEP | 28/80 | (35%) | 41/80 | (51%) | 11/80 | (13%) |
| Vancomycin | VA | 4/80 | (5%) | 76/80 | (95%) | 0/80 | (0%) |
| Amikacin | AK | 58/80 | (72%) | 18/80 | (22%) | 4/80 | (5%) |
| Gentamycin | CN | 49/80 | (61%) | 25/80 | (31%) | 6/80 | (7%) |
| Tetracycline | TE | 52/80 | (65%) | 24/80 | (23%) | 4/80 | (5%) |
| Imipenem | IMP | 44/80 | (55%) | 32/80 | (40%) | 4/80 | (5%) |
