# Supplementary material for: Multidrug resistant Klebsiella Pneumoniae reservoir and their capsular resistance genes in cow farms of district Peshawar, Pakistan
Source: PLoS One. 2023 Feb 27;18(2):e0282245. doi: 10.1371/journal.pone.0282245 (PMC9970052; doi:10.1371/journal.pone.0282245)
Supplement: S1 Raw images — (PDF) [file pone.0282245.s003.pdf]

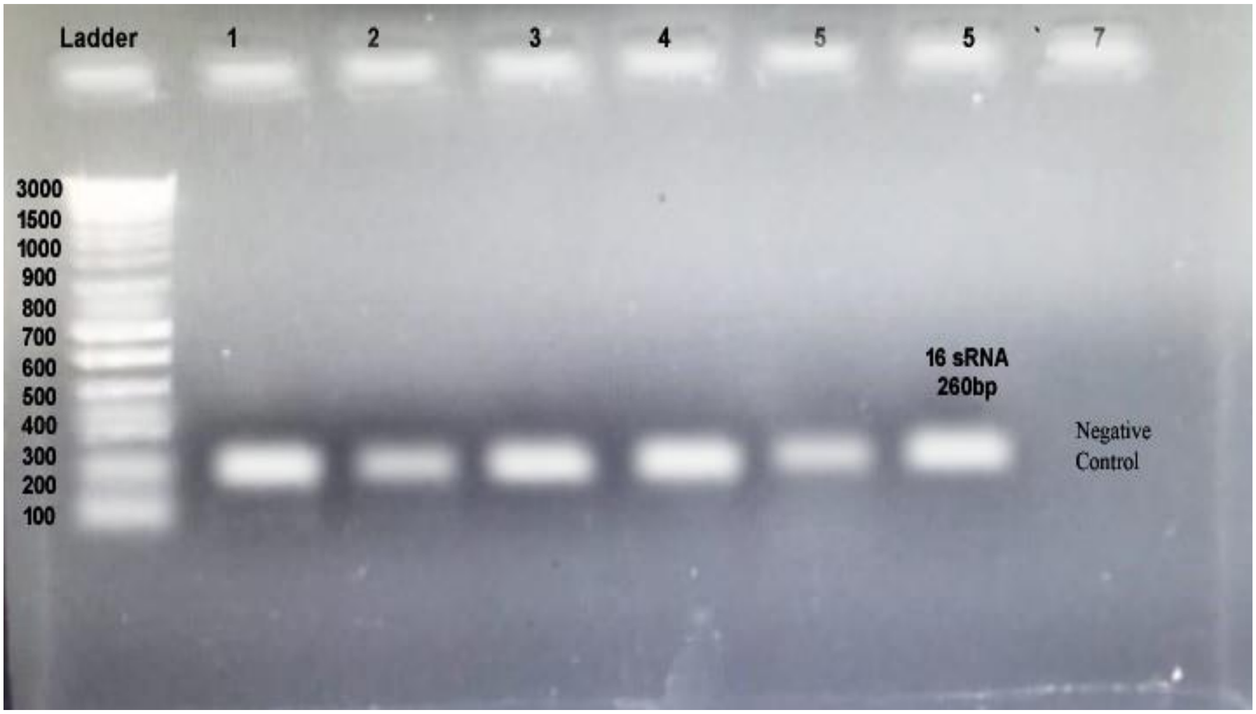

Supplementary Fig. 1. Electrophoretogram of amplified *16sRNA* gene of isolated *K. pneumoniae*

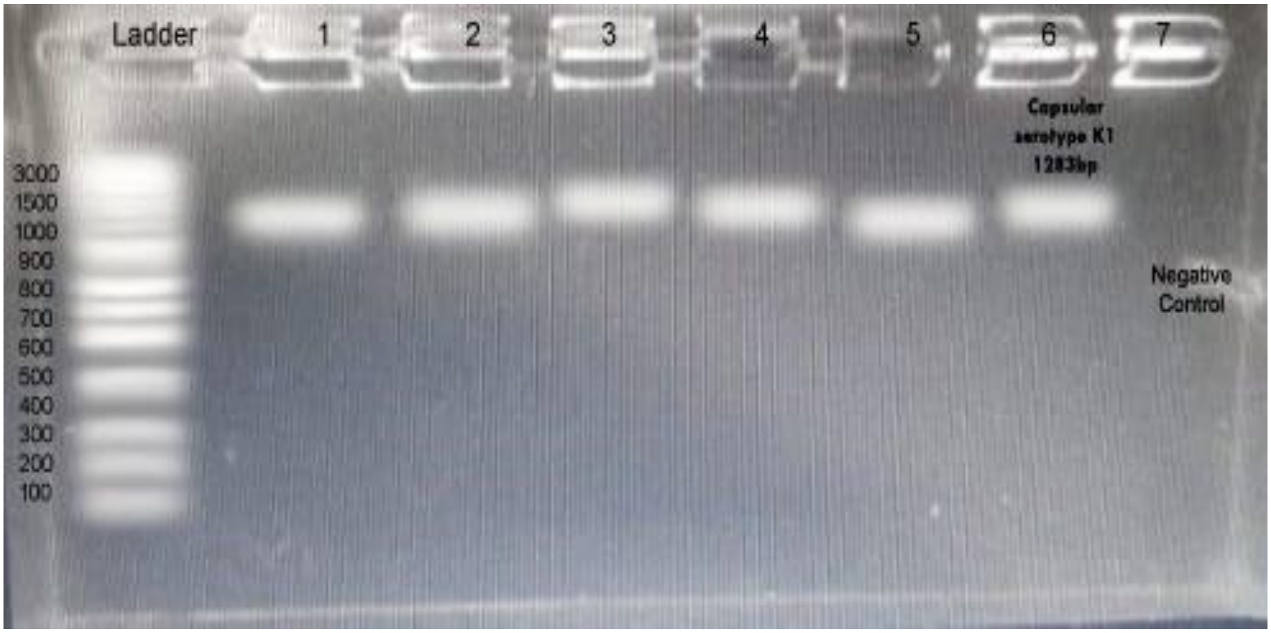

Supplementary Fig. 2. Electrophoretogram of amplified capsular resistant K1 gene from *K. pneumoniae* isolates

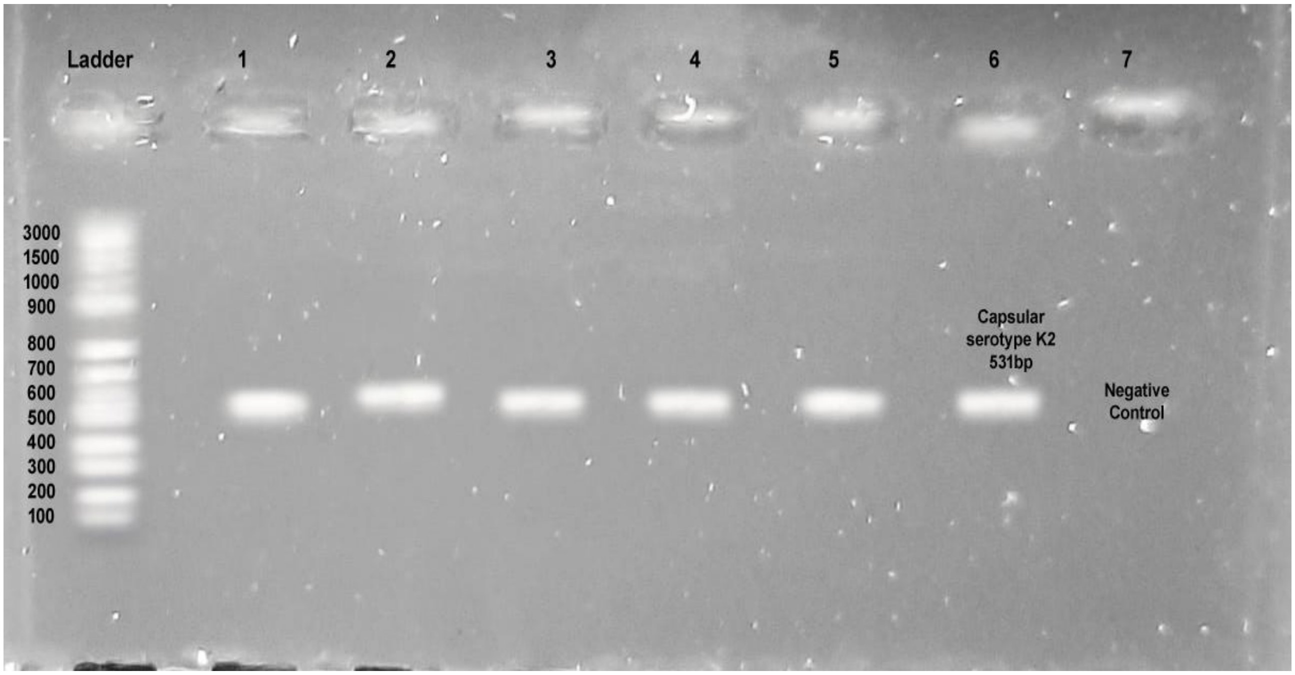

Supplementary Fig. 3. Electrophoretogram of amplified capsular resistant K2 gene from *K. pneumoniae* isolates

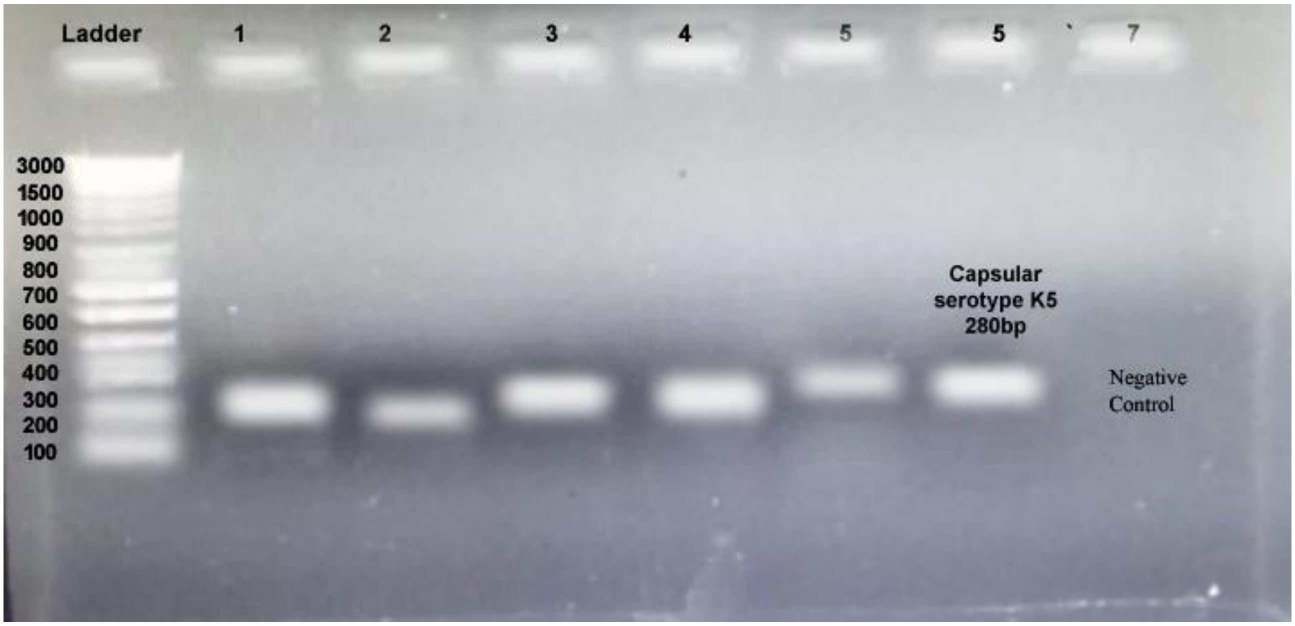

Supplementary Fig. 4. Electrophoretogram of amplified capsular resistant K5 gene from *K. pneumoniae* isolates

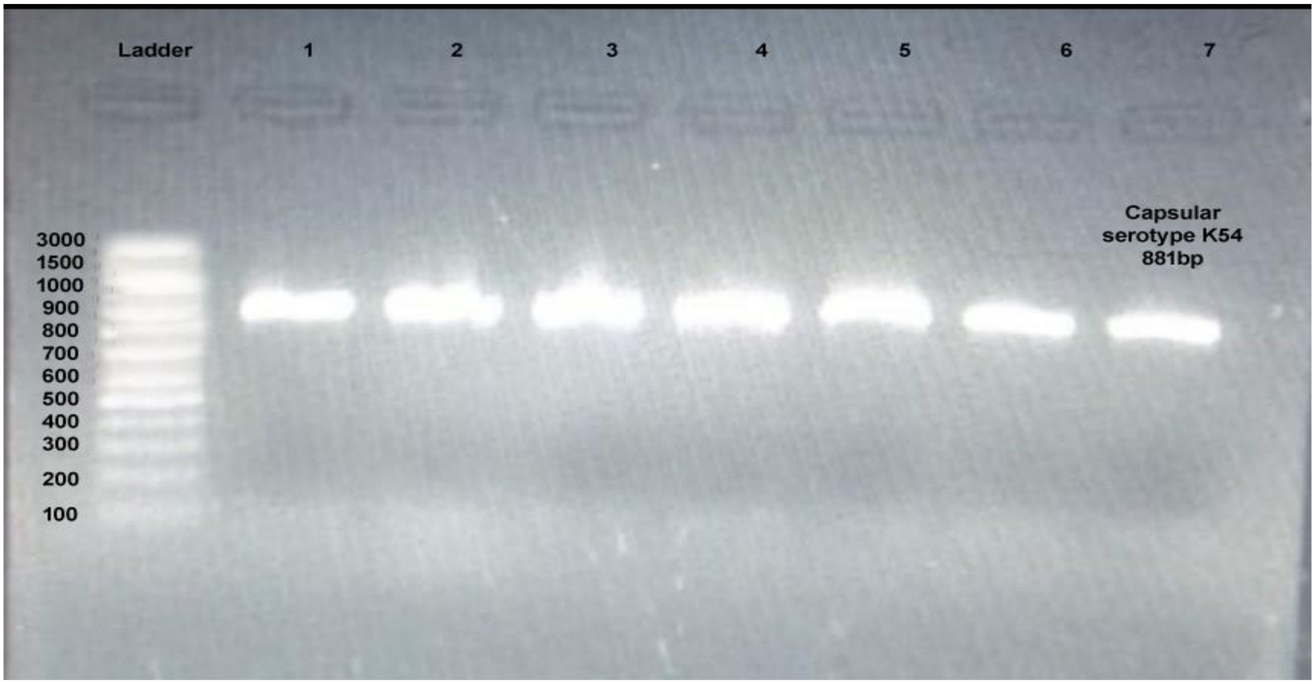

Supplementary Fig. 5. Electrophoretogram of amplified capsular resistant K54 gene from *K. pneumoniae* isolates
